# Contrast in Edge Vegetation Structure Modifies the Predation Risk of Natural Ground Nests in an Agricultural Landscape

**DOI:** 10.1371/journal.pone.0031517

**Published:** 2012-02-21

**Authors:** Nicole A. Schneider, Matthew Low, Debora Arlt, Tomas Pärt

**Affiliations:** 1 Department of Ecology, Swedish University of Agricultural Sciences, Uppsala, Sweden; 2 Centre for Agri-Environmental Research, School of Agriculture, Policy & Development, University of Reading, Reading, United Kingdom; University of Hull, United Kingdom

## Abstract

Nest predation risk generally increases nearer forest-field edges in agricultural landscapes. However, few studies test whether differences in edge contrast (i.e. hard versus soft edges based on vegetation structure and height) affect edge-related predation patterns and if such patterns are related to changes in nest conspicuousness between incubation and nestling feeding. Using data on 923 nesting attempts we analyse factors influencing nest predation risk at different edge types in an agricultural landscape of a ground-cavity breeding bird species, the Northern Wheatear (*Oenanthe oenanthe*). As for many other bird species, nest predation is a major determinant of reproductive success in this migratory passerine. Nest predation risk was higher closer to woodland and crop field edges, but only when these were hard edges in terms of ground vegetation structure (clear contrast between tall vs short ground vegetation). No such edge effect was observed at soft edges where adjacent habitats had tall ground vegetation (crop, ungrazed grassland). This edge effect on nest predation risk was evident during the incubation stage but not the nestling feeding stage. Since wheatear nests are depredated by ground-living animals our results demonstrate: (i) that edge effects depend on edge contrast, (ii) that edge-related nest predation patterns vary across the breeding period probably resulting from changes in parental activity at the nest between the incubation and nestling feeding stage. Edge effects should be put in the context of the nest predator community as illustrated by the elevated nest predation risk at hard but not soft habitat edges when an edge is defined in terms of ground vegetation. These results thus can potentially explain previously observed variations in edge-related nest predation risk.

## Introduction

In many landscapes habitat fragmentation has increased the amount of edges relative to habitat area [1], [2], [3]. Habitat edges, the boundaries between structurally different habitat types are an ongoing focus of ecological and conservation biological research because of their influence on distribution and population dynamics of many species [4], [5]. Predator-prey interactions have been shown to change substantially at habitat interfaces [1], [6], which in the case of bird communities may result in an increased nest predation risk near edges [7], [8], [9]. Thus, where species of conservation concern inhabit landscapes containing a high proportion of edges (e.g. agricultural landscapes in Europe) it is important to quantify edge effects on the population parameters of these species.

Farmland birds have declined dramatically during the last century [10], and agricultural landscapes are often highly fragmented with a high proportion of edges. In agricultural landscapes studies on edge effects have largely focused on forest interfaces and nest predation caused by avian predators e.g. [11], [12], [13]. Ground nests, however, are frequently preyed upon by mammals which often forage in edge zones or use them as movement corridors [14], [15]. What exactly constitutes an edge zone from the perspective of a predator is often unclear. Whereas an edge for an avian predator – using look-outs to find nests – may be between large structural changes such as the tree layer and open farmland habitat [16], the edge for a mammalian ground predator (mustelid, cat, fox) is likely to be dependent on smaller-scale structural changes at ground level. Thus, differences in ground vegetation structure and height can create high-contrast or ‘hard’ edges (e.g. between a mature crop field and grazed grassland) and low-contrast or ‘soft’ edges (e.g. between a mature crop field and ungrazed pasture [16]). Even forest-field edges may function differently for different suites of predators. A forest bordering on a mature crop for example may function as a hard edge for avian predators, but as a soft edge for mammalian predators, as there is continuous ground vegetation cover which tends to promote the general diffusion of small mammals from one habitat type to another [17], [18].

Edge effects, however, may not only depend on the behavioural responses of potential nest predators to the vegetation structure but also on nest conspicuousness. We thus expect the relationship between habitat edges and predation risk to vary relative to three factors: (i) the type of predator; different predators may perceive edges differently or utilize edges in different ways, (ii) the hardness of the edge (soft edges may not be perceived as edges at all), and (iii) the cues available from the nest; the level of nest conspicuousness increases through parental activity from the incubation to the nestling period and should change nest predation risk at habitat edges [19], [20]. Since these factors may interact in their effects on edge-related nest predation patterns, more knowledge is needed to draw general conclusions about the impact of edge effects on populations in fragmented landscapes, especially when an edge can shift from being hard to soft in a short space of time (i.e. seasonal growth of crops or grasslands).

A common approach to study edge effects related to nest predation has been to use an experimental design using artificial nests e.g. [11], [21]. Although such an approach has been valuable in identifying predator-prey relationships, artificial nests may not reflect actual predation risk (they do not account for parental feeding cues) which makes it difficult to disentangle the links between nest predation risk and habitat structure on real breeding attempts [22], [23]. We therefore analysed spatial and temporal nest predation patterns of 923 natural nests in a 16 year study of ground-cavity nesting Northern Wheatears (*Oenanthe oenanthe*; hereafter wheatear) breeding in a fragmented agricultural landscape. Wheatears breed in different types of farmland habitat and at a large range of distances from habitat edges, avoiding the very edge of forests [24]. The key nest predators in our system are ground-living predator species (i.e. stoat *Mustela erminea*, weasel *Mustela nivalis*, red fox *Vulpes vulpes*, Eurasian badgers *Meles meles*, domestic cats *Felis catus*, and possibly snakes) but not birds [23], [25]. The small species among these (i.e. mustelids, snakes) show a preference for tall ground vegetation and linear habitat structures, where their primary prey is more abundant (e.g. voles *Clethrionomys* spp. and *Microtus* spp. [26], [27]).

Here we investigate nest predation in relation to habitat edge type and nest conspicuousness, and their potential interactions. Specifically we were interested in answering the following three questions: (i) Can nest predation risk be explained by the structure of habitats and their edge zones, particularly edge contrast? (ii) Does edge-related nest predation risk differ with nest conspicuousness between incubation and nestling feeding?

## Methods

### Ethics statement

The permit for the study of northern wheatears was approved by the “Ethical committee of Uppsala” (Uppsala djurförsöksetiska nämnd) at the district court of Uppsala with the permit number C 117/8. The permit for ringing adults and nestlings was issued for all years by the Natural History Museeum, the bird ringing centre, with the permit number 509. All farmers within the study area personally approved us working on their land, and we thank them for their hospitality.

### Study area and population

Our study area (40 km^2^) was situated in a heterogeneous agricultural landscape south-east of Uppsala (59°50′N, 17°50′E), Sweden. This landscape consisted of a mosaic of crop fields (∼65%), woodlands (∼20%), grazed and ungrazed grasslands (∼10%) as well as farmyards and human settlements (<5%). From 1993–2008 all territories which were previously occupied or suitable for wheatears (n = 161 territories) were monitored throughout each breeding season (mid April to end of June) and classified according to land-use, habitat structure and breeding success; for details see [28], [29]. The majority of nests were on the ground under stones (∼80% in stone piles), while a small proportion of birds nested under the roof tiles on farm buildings. Egg laying started in early May, incubation lasted for about 13 days and nestlings spent about 15 days in the nest before fledging. A breeding event was defined as successful when we observed fledglings or heard intense warning calls of the parents at or after the predicted time of fledging [29]. The majority of nests were located after hatching (N = 809) and a smaller proportion during incubation (N = 114). Despite this, we were able to identify nesting success for all breeding attempts based on behavioural observations (defined by the presence of a pair over a time span of at least two weeks, i.e. nest initiation was likely to have occurred). We visited territories at least every third day and more frequently at the time of hatching. A breeding failure before the nest was found was obvious when the female changed her behaviour (visibility and activity pattern) or the pair disappeared.

Nest predation is the major cause of reproductive failure in this population, with approximately 85% of nest failures caused by predation [30] and >20% of nest predations resulting in the death of the resident female [25]. The majority of nests (70%) is depredated by small ground-dwelling predators (mustelid or snake; leaving an empty but otherwise untouched nest) with the remaining 30% being taken by large mammals (fox, badger and domestic cat; nest usually dragged or dug out; T.P. unpublished data). The proportion of nests being dragged or dug out is higher during the nestling period as compared to the incubation period (50% vs 10%, T.P. unpublished data).

Wheatears are migratory and return to the study area early in spring (mid-April) and select territories and nest sites when ground vegetation (field layer height) is generally sparse and short. However, in ungrazed areas ground vegetation grows tall during the breeding season and becomes poor foraging habitat during the late incubation and nestling period [25], [29]. Thus, we classified territories based on field layer height measurements made in regular intervals throughout the breeding season as either being permanently short (<5 cm field layer height) or growing tall (up to ≥15 cm; [29]). This classification is a good indicator of territory quality as previous studies have shown that territories with a permanently short field layers have a higher reproductive success because of higher food availability and lower nest predation risk [30], [31]. Territories with short field layers were located in grazed pastures and on farmyards where the grass layer is regularly cut, whereas territories with tall field layers were located in crop fields, leys, or unmanaged grassland [28].

### Definition of edges

We categorised habitat edges in our study according to the edge contrast concept [16] put in relation to nest predator behaviour, which differentiates edges according to the contrast in vegetation height and density between adjacent habitat types. Differences in edge responses will thus mainly be due to differences in the permeability of the edge [16], with weaker effects near ‘soft’ (low-contrast) edges than near ‘hard’ (high-contrast) edges [5]. In our case soft or low-contrast habitat edges were defined as adjacent habitats that both feature either tall or short ground vegetation; e.g. mature crop field (tall) – ungrazed pasture (tall), mature crop field (tall) – woodland (tall), or grazed pasture (short) – farmyard (short). Hard or high-contrast habitat edges were defined as adjacent habitats where one habitat has short ground vegetation and the other tall; e.g. grazed pasture (short) – mature crop field (tall), grazed pasture (short) – woodland (tall), or newly-sown crop (short) – mature crop field (tall).

### Digital mapping and data selection

We digitalised the distribution of land-use patterns in our study area (i.e. grassland, crop, woodland, buildings, roads and pathways) based on aerial photographs (Lantmäteriet 1999) in ArcGIS 9.2 (ESRI 2006). We also digitised the location of all ground-level wheatear nests where the exact location of the nest and outcome of the breeding attempt was known from 1993–2008. We excluded breeding failures caused by events other than predation, such as starvation or nests drowned during heavy rain, leaving us with 923 nests for the analysis. Around each nest we created a buffer with an 80 m radius, resulting in an area of about 2 ha which corresponds to the average breeding territory size of wheatears in pasture areas (T.P. unpublished data). Within each buffer we measured: (i) the area of woodlands, crop fields, and grasslands, (ii) the length of linear habitats like road verges and habitat edges of woodlands, crop fields, and grasslands, and (iii) the nearest distance from the nest to habitat edges, road verges, and buildings (higher rodent densities on farms may attract predators and pose a greater risk of domestic cat predation).

### Statistical analyses

We used generalized linear mixed models (GLMM) with logit link binominal error structure and Laplace parameter estimation in R 2.8.1 [32] to analyse the relationship between nest predation risk (survive or fail) and habitat variables. We repeated this analysis to separately analyse the factors influencing nest predation risk during incubation (N = 923) and nestling feeding (N = 839). The GIS-generated variables were highly correlated (r≥0.7; i.e. habitat area to edge length, or edge length to edge distance). Thus, we only used distance measures from nest locations to habitat elements in the analyses. We chose distance measures over area and edge length since these variables unify edge and area effect aspects, i.e. nests closer to linear habitat elements should experience a greater edge effect and nests further out in a habitat patch will be located within a larger patch. As fixed effects we included the distance from the nest to the nearest woodland, crop field, road, and houses – as well as field layer height between the edge and the nest (short or tall) and the two-way interactions between field layer height and the distance variables. We kept all main effects in the models but dropped all interactions p>0.05. Year and territory identity were fitted as crossed random effects to control for repeated samplings of nest predation risk within and between years.

Since the above analyses only investigated nest survival when the exact location of the nest was known, habitat-specific nest predation risk estimates could be biased due to the exclusion of early nest failures. Nests in high-risk areas are more likely to fail before their location is known and these early failures might not be uniform with respect to field layer height (since field layer height significantly influenced nest predation risk in the above incubation and nestling stage models). To account for this, we modelled survival for the different nest stages for all ground nests in short and tall field layer habitats from 1993–2008 regardless whether the nest location was exactly known (N = 1235; consisting of known nest location N = 923, not exactly known nest location N = 312). We used Cormack-Jolly-Seber live-recapture models in program MARK [33]. Each nest's encounter history consisted of three time intervals (incubation, nestling period, post fledging) resulting in two survival periods: (1) from laying to hatching, and (2) from hatching to fledging (18 days based on modal clutch size of 6 and 15 days nestling period [24]). Survival is expressed in units of daily survival rates as the nest stages were of different length. We used this approach rather than a typical nest survival analysis e.g. [34] because we could not always accurately estimate nest age beyond a simple incubation/nestling-stage dichotomy. Our approach is valid in this case because we were able to monitor all pairs in the study area and had enough adult behavioural observations to classify all pairs as failing during incubation, failing during the nestling period or successfully fledging young. Thus, there was no bias from undetected nesting failures. By manipulating the parameter index matrices in program MARK [35] we could vary or constrain the four survival parameters of interest (incubation period in short field layer habitats, nestling period in short field layer habitats, incubation period in tall field layer habitats, and nestling period in tall field layer habitats) to test hypotheses regarding differences in nest survival between nesting phases (incubation and nestling) and between habitat vegetation type (tall and short field layer height). We compared four models: (i) same survival between the four groups, (ii) survival varying between all four groups, (iii) survival only varying between habitat types, (iv) survival only varying between nesting stages. We used Akaike's information criterion with a second-order correction for sample size (AIC_c_), with the strength of support for each model being based on its AIC weight (*w_i_*). Resighting-probability was set to one for all analyses and reported estimates for the survival parameters are derived from model averaging across all candidate models.

## Results

### Habitat structure and nest predation risk

**Figure 1:**
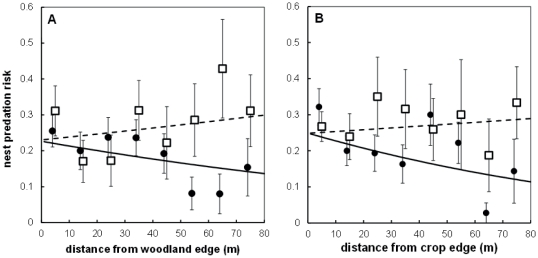
Nest predation risk during the breeding season (incubation+nestling stage) in short field layer (black) and tall field layer (dashed) breeding territories in relation to: (a) distance from woodland edge, and (b) distance from crop field edge. Lines show model predictions; points raw data (mean ± SE).

Of the 923 ground nests with known nest location 206 were predated (84 during incubation and 122 during the nestling stage). Analysing all nests independent of nesting stage nests closer to woodland or crop field edges had a higher predation risk than those farther away (Table 1). This relationship depended on the height of the field layer surrounding the nest (Table 1; Figure 1a, b). For nests within a short field layer habitat (e.g. grazed pasture) predation risk increased the closer they were to woodland edges (i.e. hard edge), whereas for nests within tall field layers (i.e. soft edge) predation risk did not increase closer to woodlands (Figure 1a). We found a similar pattern for distance to mature crop field edges. Nests within short field layers had a higher predation risk the closer they were located from crop field edges (i.e. hard edge), while nest predation risk in tall field layer habitats (i.e. soft edge) showed no such distance relationship (Figure 1b). The predation risk of wheatear nests did not depend on the distance to buildings or road verges (Table 1).

**Table 1:** Model (binomial GLMM) on nest predation risk of wheatear nests (n = 923) in relation to different habitat structures.

Fixed effects	estimate	se	z-value	p-value
**Intercept**	0.907	0.342	2.9	0.003
Woodland distance	−0.018	0.009	1.9	0.044
Crop distance	−0.025	0.009	2.8	0.005
House distance	0.005	0.004	−1.3	0.188
Road distance	0.001	0.003	−0.4	0.675
FLH	−0.575	0.381	1.5	0.131
FLH * woodland distance	0.011	0.006	−1.9	0.051
FLH * crop distance	0.014	0.006	−2.4	0.014

Habitat structures = distance from the nest woodland or crop edge, and field layer height (FLH) around the nest site (tall or short; reference category = short). Year and territory identity were included as crossed random effects (variance of year = 0.17, territory = 0.16). Dropped non-significant interaction terms: FLH*house distance p = 0.36; FLH*road distance p = 0.93.

During incubation, nest predation risk increased closer to woodland and crop field edges (Table 2) and was higher for nests surrounded by tall field layer habitats as compared to nests surrounded by short field layer habitats. During the nestling feeding period nest predation risk was only associated with field layer height surrounding the nest but not significantly linked to any of the habitat edge variables investigated (Table 2).

**Table 2:** Model (binomial GLMM) on nest predation risk during the incubation (n = 923) and nestling period (n = 839) in relation to different habitat structures.

Fixed effects	estimate	se	z-value	p-value
**Incubation period**				
Intercept	1.712	0.496	3.4	0.0005
Woodland distance	−0.009	0.005	1.7	0.092
Crop distance	−0.017	0.006	2.9	0.004
House distance	0.005	0.005	−0.9	0.323
Road distance	−0.002	0.004	0.4	0.718
FLH	−1.248	0.523	2.4	0.011
FLH * woodland distance	0.019	0.008	−2.3	0.023
FLH * crop distance	0.020	0.008	−2.5	0.013
**Nestling period**				
Intercept	2.417	0.457	5.3	<0.0001
Woodland distance	−0.002	0.004	0.5	0.61
Crop distance	−0.003	0.004	0.7	0.46
House distance	0.004	0.005	−0.9	0.36
Road distance	0.003	0.004	−0.7	0.46
FLH	0.502	0.235	−2.1	0.03

Habitat structures = distance from the nest to woodland or crop edge, and field layer height (FLH) around the nest site (tall or short; reference category = short). Year and territory identity were included as crossed random effects (Incubation: variance year = 0.14, territory = <0.0001; Nestling: variance year = 0.31, territory = 0.38). Dropped non-significant interaction terms incubation stage model: FLH*house distance p = 0.85; FLH*road distance p = 0.44. Dropped non-significant interaction terms nestling stage model: FLH*woodland distance p = 0.65; FLH*crop distance p = 0.35; FLH*house distance p = 0.13; FLH*road distance p = 0.55.

### Daily nest survival during incubation and nestling feeding

**Figure 2:**
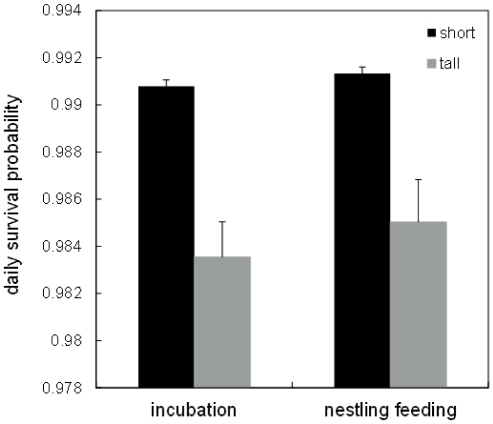
Daily survival probability (± SE) of 1235 wheatear nests during the incubation and nestling phase in short field layer (short) vs. tall field layer (tall) habitats. Evidence of predation on nests with an unknown exact location within the territory (N = 312 out of 1235) was based on behavioural observations (see methods).

We analysed daily nest survival, including also early nest failures for which we could not locate the exact nest position (nest outcomes based on observations of adult behaviour, see Methods), to test whether there was a differences in habitat-specific nest predation risk between the incubation and nestling period and in territories having tall or short field layers. Of 1235 nests 333 were predated (187 during incubation and 146 during the nestling stage). Daily nest survival probability (Φ) differed between habitat vegetation structure (tall vs short field layer habitats: 0.984±0.001 vs 0.991±0.001), with this difference being the equivalent of a 59% nesting success in tall habitats and 74% in short habitats. There was strong support for habitat-related difference in nest survival and little support for survival differences between incubation and the nestling period (Φ_habitat_ ΔAIC_c_ = 0.0, AIC_c_ weight (*w_i_*) = 0.85; Φ_habitat, nesting_stage_ ΔAIC_c_ = 3.5, *w_i_* = 0.15; Φ_constant_ ΔAIC_c_ = 28.5, *w_i_* = 0; Φ_nesting_stage_ ΔAIC_c_ = 29.7, *w_i_* = 0). Thus within each habitat type, daily nest survival was similar for both the incubation and the nestling period (Figure 2).

## Discussion

It has been long established that habitat edge structures can substantially influence predator dynamics e.g. [36], but relatively few studies have investigated the effects of edge contrast on nest predation risk [16]. Previous investigations into nest predation at different habitat edges have shown that nest predation risk is higher at hard habitat edges within forested landscapes [37], [38]. These findings, however, relied on artificial nest experiments which may not reflect real predation risk [22]. Using data from 923 natural nests we show that in an agricultural landscape nest predation risk in proximity to habitat edges is context dependent. Nests located close to a hard edge from the predator perspective (i.e. short-tall ground vegetation boundary) had an increased nest predation risk whereas this effect was absent for nests located close to a soft edge. This effect of hard edge was only detectable during incubation, while during nestling feeding nest predation risk was exclusively determined by the field layer height of the habitat surrounding the nest site. Thus, our results partly support previous findings from artificial nests studies, but show that edge-related nest predation risk may be linked to edge type and nesting stage.

Previous studies based on artificial nests investigating edge effects on nest predation risk suggest higher predation risk along hard as compared to soft edges e.g. [38], possibly because predator activity is spatially more concentrated along hard edges. These patterns are consolidated by our results on the predation of natural nests and are in agreement with the behaviour of nest predator species. The most common predators of wheatear nests (mustelids, snakes) usually show higher density and activity along habitat edges and linear structures such as ditches, road verges or field edges [27], [39]. Mustelids prefer tall vegetation and rarely travel far from linear elements [46] because of the higher abundance of their primary prey (i.e. voles) and greater cover from intraguild predation by aerial hunting raptors [39], [40]. In our study area vole abundance and activity is higher along hard habitat edges and linear structures (footprint tracking tunnel study [41]). Thus, the activity of the main nest predators is most likely concentrated in tall field layer habitats and along hard edges.

The higher nest predation risk of wheatear nests in tall field layer habitats at greater distances from woodland edges seems to contradict other studies which found higher nest predation levels and predator abundances close to forest and woodland edges [8], [42]. This relationship could be explained by the behaviour of predators and their primary prey. As crop and ungrazed grassland height increases during spring, voles migrate into the fields from the habitat edges which they occupied during winter and reach higher densities within tall field layer areas than within edge habitats [43], [44]. If mustelids follow their main prey and also migrate from the edges into the tall vegetation, the level of incidental nest predation risk should increase in these areas [45].

Edge effects were only apparent during the incubation period. This may initially seem surprising, as nest conspicuousness increases in the nestling period due to parental activity during nestling provisioning [20], [46]. Thus, one should expect nest predation risk to increase over the nesting cycle. Such a pattern, however, was not supported by our data. Nests generally were more likely to be predated when located in tall field layer habitats, both during incubation and nestling feeding, with no change in nest predation probability between nesting stages. One explanation for this pattern could be that nests located in high-risk areas are more likely to be preyed upon first and are disproportionately taken during the incubation period. This would balance out increases in nest predation risk associated with increased nest conspicuousness during nestling feeding [20], [47].

The absence of an edge effect during the nestling stage can be due to two mutually non-exclusive explanations. (i) A change in predator composition that goes along with a change in spatial predator activity. Wheatear nests, like the nests of other ground breeding bird species, are very cryptic throughout the incubation period and offer only few cues for active nest detection. Nest predation during incubation is mainly caused by predators like mustelids (see Methods, T.P. unpublished data) which find nests incidentally while hunting for their primary prey (i.e. voles). Predation patterns during the incubation stage should thus mainly reflect spatial predator activity. The abundance of nest activity related cues during nestling feeding expands the spatial range at which nests can be detected to greater distances from edges and cover. As a consequence, small scale patterns of edge-related predation risk are likely to disappear. In line with this suggestion is that predation by large mammals predominantly occurs at the nestling stage (see Methods). These large predators, as well as mustelids, can use longer-range visual and acoustic cues to detect nest activity from a distance [48], which might explain why edge effects on predation risk were only apparent during incubation in our study. (ii) Seasonal growth of field layer height transforms a proportion of initially hard edges into soft edges when wheatears are feeding nestlings. Thus, edges that soften through the growth of adjacent vegetation (e.g. between a crop field and cultivated grassland) can cease being edges at all [7], [16]. From the perspective of many ground-based predator species a lack of change in the ground vegetation structure at habitat interfaces may thus not represent edges but a continuous landscape of tall vegetation cover [39].

Our study based on natural nests demonstrates that the relationship between nest predation risk and habitat edges can be highly variable and depend on the ‘hardness’ of the edge and the stage of the breeding cycle. This variation in the relationship between nest predation risk and habitat edges can be understood in terms of nest predator species and their behaviour, seasonal changes in vegetation structure and nest conspicuousness, highlighting the complex interplay of factors affecting avian nest predation risk in fragmented landscapes. This suggests some limitations in general approaches that only look at habitat interfaces without accounting for predator behaviour [42], [49] or only one breeding stage (e.g. often the nestling stage is studied for natural nests and the egg stage for artificial nests). Since mammals are among the main predators of ground nesting birds in agricultural landscapes [14], [15], the effects of ground vegetation edge contrast on nest predation risk should be of relevance for the breeding success of ground nesting farmland birds in general. Further investigations on changes in nest predation patterns over the nesting cycle and temporal changes in edge contrast should thus be of importance in agricultural landscapes featuring many edges and a high variability in vegetation height. Since many farmland and grassland bird species are declining, a better understanding of nest predator-habitat interactions is important for the implementation of effective conservation measures.
